# Supplementary material for: Queuosine biosynthetic enzyme, QueE moonlights as a cell division regulator
Source: PLoS Genet. 2024 May 20;20(5):e1011287. doi: 10.1371/journal.pgen.1011287 (PMC11142719; doi:10.1371/journal.pgen.1011287)
Supplement: S1 Text — Table A. List of strains used in the study. Table B. List of plasmids used in the study. Table C. List of oligonucleotides used in the study. Fig (i). His6-tagged QueE regulates septation in a dose-dependent manner. (a) Measurement of cell lengths of E. coli MG1655 ΔlacZYA (TIM183) cells expressing QueE. Strains containing a plasmid encoding E. coli His6-EcQueE (pSY85) or empty vector (pEB52) were grown in supplemented MinA minimal medium for 2 hours (OD600 = 0.2–0.3) and induced with IPTG for 3 hours at the indicated concentration. Gray circles represent individual cells, mean cell length values are indicated in red, and the horizontal gray bars represent the median. Data are obtained from four independent experiments using the number of cells indicated by (n) for each inducer concentration. Statistical analysis was done using t-test displaying significance at *P ≤ 0.05, **P ≤ 0.01, ***P ≤ 0.001, ****P ≤ 0.0001, and “ns” = P >0.05 (b) Representative phase-contrast micrographs of ΔlacZYA cells expressing QueE at the indicated inducer concentration, scale bar = 5 μm. (c-d) Representative Coomassie gel showing protein levels in total cell lysates at different IPTG concentrations and the corresponding western blot showing His6-QueE levels. e) APB gel-northern blot detecting tRNATyr in total RNA samples of ΔqueE (SAM31) cells encoding either WT QueE (pRL03), His6-QueE (pSY85) or an empty vector (pEB52). Cells were grown in supplemented MinA minimal medium for 2 hours and induced with 0.5 mM IPTG for 3 hours. G-tRNATyr = unmodified tRNATyr, Q-tRNATyr = Q-modified tRNATyr. Note that this panel is a montage with the data for pHis6-QueE acquired on another blot, as indicated with a dotted line. (f) Plot showing the relationship between average cell lengths (from panel a) and relative QueE abundance (from panel d), see methods for details. Fig (ii). Alanine scanning mutagenesis of amino acid residues in EcQueE important for Q biosynthesis. (a) APB northern blot detecting tRN [file pgen.1011287.s001.docx]

**Supporting Information**

# **Tables**

##

## **Table A. List of strains used in the study**

| Strain | Relevant genotype | Reference/source |
| --- | --- | --- |
| MG1655 | *λ^-^ rph^-1^* | *E. coli* Genetic Stock Center, CGSC no. 7740 |
| SAM31 | MG1655 *ΔqueE* λ_att_ (P_mgrB_-*yfp*) HK_att_ (P_tetA_-*cfp*) | Goulian lab |
| SAM54 | MG1655 *ΔqueE* MG1655 Φ(*queE^+^*  *-yfp^+^*) KanR | [1] |
| SAM96 | MG1655 *ΔqueE* | [1] |
| TIM183 | MG1655 Δ*lacZYA* |  |
| Top10 | *F– mcrA Δ(mrr-hsdRMS-mcrBC) φ80lacZΔM15 ΔlacX74 recA1 araD139 Δ(ara-leu)7697 galU galK λ– rpsL (StrR) endA1 nupG* | Invitrogen |
| XL1-Blue | *recA1 endA1 gyrA96 thi-1 hsdR17 supE44 relA1 lac [F´ proAB lacIqZΔM15 Tn10 (Tetr)]* | [2] |
| *S. Typhimurium* *14208* | *Salmonella enterica subsp. enterica* (ex-Kauffmann and Edwards) Le Minor and Popoff serovar Typhimurium, ATCC 14028 | Schifferli lab |
| Klebsiella pneumoniae 13883 | *Klebsiella pneumoniae subsp. pneumoniae* (Schroeter) Trevisan 13883 | Fan lab |
| *P. aeruginosa* PAO1 | *lacI^q+^* *Δ*(*lacZ*)M15^+^ *tetA*^+^ *tetR*^+^ | Nickels lab |
| *B. subtilis* 168 | *trpC2* | Boyd lab |
| *B. subtilis* IS75 | His^-^ Leu^-^ Met^-^, *amyE*^+^ | Dubnau lab |
| SAA50 | *B. subtilis* IS75 ∆*amyE*::pSA147 (*BsqueE*) | This work |
| SAA52 | *B. subtilis* IS75 *∆amyE*::pSA149 (*yfp-BsqueE*) | This work |
| SAA53 | *B. subtilis* IS75 *∆amyE*::pPDR111 (*empty*) | This work |

##

## **Table B. List of plasmids used in the study**

| Name | Description | Reference/source |
| --- | --- | --- |
| pBAD33 | ori(p15A), ParaBAD-MCS araC, camR | ATCC 87402 |
| pDR111 | amyE-hyper-SPANK, specR, carbR. | Dubnau lab |
| pEB52 | pTrc99a with the NcoI site removed, carbR | Goulian lab |
| pEG4 | pBAD33 ftsZ-mCherry, camR | [3] |
| pPSV38 | Shuttle vector, *lacI^q^*, *lacUV5* promoter,  origins of replication, *colE1* and   pRO1600*,* gentR | Dove lab |
| pRL03 | pEB52-EcQueE carbR | [1] |
| pSA1 | pEB52-EcQueE C31A, carbR | This work |
| pSA2 | pEB52-EcQueE C35A, carbR | This work |
| pSA3 | pEB52-EcQueE C38A, carbR | This work |
| pSA5 | pEB52-EcQueE E15A, carbR | This work |
| pSA8 | pEB52-EcQueE S136A, carbR | This work |
| pSA9 | pEB52-EcQueE K60A, carbR | This work |
| pSA10 | pEB52-EcQueE K66A, carbR | This work |
| pSA11 | pEB52-EcQueE K194A, carbR | This work |
| pSA21 | pEB52-StQueE, carbR | This work |
| pSA22 | pEB52-PaQueE, carbR | This work |
| pSA55 | pEB52-EcQueE W67A, carbR | This work |
| pSA57 | pEB52-yfp-EcQueE K66A, carbR | This work |
| pSA58 | pEB52-yfp-EcQueE R27A, carbR | This work |
| pSA59 | pBAD33-KpQueE, camR | This work |
| pSA60 | pRL03-QueE ∆E45-E51, carbR | This work |
| pSA61 | pRL03-QueE ∆V52-W67, carbR | This work |
| pSA62 | pRL03-QueE ∆E45-W67, carbR | This work |
| pSA73 | pEB52-yfp-EcQueE E15A, carbR | This work |
| pSA75 | pEB52-yfp -EcQueE C31A, carbR | This work |
| pSA76 | pEB52-yfp-EcQueE ∆E45-W67 | This work |
| pSA119 | pEB52-yfp-QueE C35A, carbR | This work |
| pSA121 | pEB52-yfp-pBsQueE, carbR | This work |
| pSA126 | pEB52-yfp-pPaQueE, carbR | This work |
| pSA147 | pDR111-BsQueE, specR, carbR. | This work |
| pSA149 | pDR111-yfp-BsQueE, specR, carbR. | This work |
| pSA151 | pUCP20T-PaQueE, carbR | This work |
| pSA154 | pPSV38-yfp-PaQueE, gentR | This work |
| pSA156 | pPSV38-PaQueE, gentR | This work |
| pSA157 | pPSV38-EcQueE, gentR | This work |
| pSY45 | pEB52-BsQueE, carbR | This work |
| pSY76 | pEB52-yfp-QueE, contains a strong ribosome binding site (AAAGAGGAGA), no IPTG induction required, carbR | [1] |
| pSY85 | pEB52-His_6_-QueE, carbR | This work |
| pSY97 | pEB52-EcQueE Q13A, carbR | This work |
| pSY98 | pEB52-EcQueE R27A, carbR | This work |
| pSY99 | pEB52-EcQueE T40A, carbR | This work |
| pSY100 | pEB52-EcQueE Q189A, carbR | This work |
| pTrc99a | Expression vector, pBR322 ori, pTrc promoter, *lacI*^q^, carbR | [4] |
| pUCP20T-eyfp | Shuttle vector encoding YFP, *lacUV5*  promoter, origins of replication, *colE1* and  pRO1600, carbR | Addgene #78466 |

carbR = carries a carbenicillin/ampicillin-resistance marker; camR = carries a chloramphenicol-resistance marker

##

## **Table C. List of oligonucleotides used in the study**

| Name of Primer | 5ʹ - 3ʹ | Reference/source |
| --- | --- | --- |
| SA1 | TACAGTCTGCTCCCTTTGGCCGCTCGGGAACCCCAC | Probe for tyrU gene |
| SA3F | GCCCCGGTTGGCTGTGCCTG | Inv PCR Primer for C31A |
| SA3R | TCCCTGTAAACGAATAAAAATGGCGG | Inv PCR Primer for C31A |
| SA4F | GCCGCCTGGTGCGACACCAAACAC | Inv PCR Primer for C35A |
| SA4R | GCCAACCGGGCATCCCTGTAAACG | Inv PCR Primer for C35A |
| SA7F | ACCTTGCAGGGTCTGGAACATCTCG | Inv PCR Primer for E15A |
| SA7R | GCCGGTTACTTTACCGGCGTTCCCG | Inv PCR Primer for E15A |
| SA9F | GCCGAGCCTTGCATTCATGATTTGCTGCC | Inv PCR Primer for G94A |
| SA9R | ACCCGTAATCACCACATGCCGC | Inv PCR Primer for G94A |
| SA11F | GTTGGCTGTGCCTGGGCCGACACCAAACACAC | Single Primer SDM for C38A in *E. coli* QueE |
| SA13F | GCCTGGGGGGCTGCGAGCAGTG | Inv PCR Primer for K66A |
| SA13R | ATCACTCTCTTTGGTCTTCGCCAG | Inv PCR Primer for K66A |
| SA12F | GCCACCAAAGAGAGTGATAAGTGGG | Inv PCR Primer for K60A |
| SA12R | CGCCAGAATGCTGAAAAGGGA | Inv PCR Primer for K60A |
| SA10F | GCCCCAAAGCTGAACATGCGCGGC | Inv PCR Primer for S136 |
| SA10R | TACGGTAACCCAGGTATTCGGTGTGC | Inv PCR Primer for S136 |
| SA5F | GCCGACACCAAACACACCTGGG | Inv PCR Primer for C38A |
| SA5R | CAGGCACAGCCAACCGGGC | Inv PCR Primer for C38A |
| queE-Q189A-L1 | CAGTGCAATGACTCGCGGTTTATCATC | Inv PCR Primer for Q189A |
| queE-Q189A-U1 | gcgCCGATTAGCCAAAAGGATGATGCCA | Inv PCR Primer for Q189A |
| queE-T40A-L1 | GTCGCACCAGGCACAGCCAAC | Inv PCR Primer for T40A |
| queE-T40A-U1 | gcgAAACACACCTGGGAAAAGCTTGAG | Inv PCR Primer for T40A |
| queE-R27A-L1 | AATAAAAATGGCGGGAACGCCG | Inv PCR Primer for R27A |
| queE-R27A-U1 | gcgTTACAGGGATGCCCGGTTGG | Inv PCR Primer for R27A |
| queE-Q13A-L1 | CAGGGTCTGGAACATCTCGTTAATCG | Inv PCR Primer for Q13A |
| queE-Q13A-U1 | gcgGGTGAGGGTTACTTTACCGGC | Inv PCR Primer for Q13A |
| SA79F | GCGTTACAGGGATGCCCGGTTGG | R27A F-primer |
| SA79R | AATAAAAATGGCGGGAACGCCG | R27A R-primer |
| SA80F | aaattaagcATGCAGTACCCGATTAACGAGATG | Adds RBS to pSA56 |
| SA80R | ctcctctttaatGgtaccgagctcgaattcg | Adds RBS to pSA56 |
| SA26R | CATCCGCCAAAACAGCCAAG | Seq R-Primer for pEB52 inserts |
| Bs_queE-SacI-U1 | GACTACGAGCTCAAAAGGGTGGTTTGAATGGCTAAAG | BsQueE in pEB52 |
| Bs_queE-BamHI-L1 | AGTAGTGGATCCCTATTATACTCCGCGTTTGTTGCC | BsQueE in pEB52 |
| SA28F | CAGAGAATTCatgGTTTCAGCTCGGCTGATCTTC | StQueE in pEB52 |
| SA28R | TAGAGGATCCtcaATGGCCCGGTTCGTCG | StQueE in pEB52 |
| SA29F | CAGAGAATTCATGCAGTACCCGATTAACGAAATGTTCC | PaQueE |
| SA29R | TAGAGGATCCTCAGGCGATATTTAAATACTTATGCGTTTGC | PaQueE |
| SA78F | ATTAGGTACCATGCAGTACCCGATTAACGAGATGTTC | KpQueE in pBAD33 |
| SA78R | ATTATCTAGATCAGGCAATATTCAGATACTTGTGCGTC | KpQueE in pBAD33 |
| SA81F | ggcagcggcGTCTCCCTTTTCAGCATTCTGGCG | Inverse PCR Primer to remove E45-E51 to pRL03 |
| SA81R | gctgccgccCCAGGTGTGTTTGGTGTCG | Inverse PCR Primer to remove E45-E51 to pRL03 |
| SA82F | ggcagcggcGGGGCTGCGAGCAGTGAAG | Inverse PCR Primer to remove V52-W67 to pRL03 |
| SA82R | gctgccgccTTCCCGATCCTCAAGCTTTTCCC | Inverse PCR Primer to remove V52-W67 to pRL03 |
| SA69F | Ccataagattagcggatcctacctgac | pBAD33 Forward sequencing primer |
| SA69R | tcagaccgcttctgcgttc | pBAD33 Reverse sequencing primer |
| SA125R_pSY76_R | tctagtTTTGTATAGTTCATCCATGCCATGTG | Hifi assembly vector primer |
| SA125F_pSY76_Bs | CAACAAACGCGGAGTAtaaTTAAACATTTATAAGCGTTATAAATGGGTGGAACC | Hifi assembly vector primer for BsQueE |
| SA125F_pSY76_Pa | GAACCGGGCCATtgaTTAAACATTTATAAGCGTTATAAATGGGTGGAACC | Hifi assembly vector primer for PaQueE |
| SA126F_Bs_F | CATGGATGAACTATACAAAactagaatgGCTAAAGGAATTCCTGTATTAGAAATTTTCGG | Hifi assembly insert primer for BsQueE |
| SA126R_Bs_R | ttaTACTCCGCGTTTGTTGCCC | Hifi assembly insert primer for BsQueE |
| SA127F_Pa_R | TTAAtcaATGGCCCGGTTCGTC | Hifi assembly insert primer for PaQueE |
| SA127R_Pa_Corr_F | CATGGATGAACTATACAAAactagaatgCAACAGACCCTGCGC | Hifi assembly insert primer for PaQueE |
| \| SA149_  BsQueE_F \| \| --- \| | TATATGTCGACTGAAAGGAGAGGGAATCAAATTGGCTAAAGGAATTCCTGTATTAGAAATTTTCG | Cloning BsQueE with a Sal1 cut site. |
| \| SA149_  BsQueE_R \| \| --- \| | TATATGCTAGCTTATACTCCGCGTTTGTTGCCC | Cloning BsQueE with a Nhe1 cut site |
| \| SA150_YFP_  BsQueE_F \| \| --- \|   SA1 | TATATGTCGACTGAAAGGAGAGGGAATCAAATTGCGTAAAGGAGAAGAACTTTTCACTGG | Cloning YFP-BsQueE with a Sal1 cut site |
| SA157R | CTGGCGAAAGGGGGATGTG | Forward Sequencing primer for pUCP20T vector and its derivatives |
| SA157F | CACTTTATGCTTCCGGCTCGTATG | Reverse Sequencing primer for pUCP20T vector and its derivatives |
| SA155F | CTAGAATTAAAGAGGAGAAATTAAGCATGCAACAGACCCTGCGC | Forward primer to clone PaQueE into pUCP20T |
| SA154R | CCAAGAAGGTCTAGAATTAAAGAGGAGAAATTAAGCATGCGTAAAGG | Reverse primer to clone PaQueE into pUCP20T |
| SA157R | CTGGCGAAAGGGGGATGTG | Forward Sequencing primer for pSA151 |
| SA157F | CACTTTATGCTTCCGGCTCGTATG | Reverse Sequencing primer for pSA151 |
| SA25F | TCGTATAATGTGTGGAATTGTGAGCG | Forward Sequencing primer for pSA126 |

**Figures**

**Fig (i). His_6_-tagged QueE regulates septation in a dose-dependent manner.** (a) Measurement of cell lengths of *E. coli* MG1655 ∆*lacZYA* (TIM183) cells expressing QueE. Strains containing a plasmid encoding *E. coli* His_6_-*Ec*QueE (pSY85) or empty vector (pEB52) were grown in supplemented MinA minimal medium for 2 hours (OD_600_ = 0.2-0.3) and induced with IPTG for 3 hours at the indicated concentration. Gray circles represent individual cells, mean cell length values are indicated in red, and the horizontal gray bars represent the median. Data are obtained from four independent experiments using the number of cells indicated by (n) for each inducer concentration. Statistical analysis was done using t-test displaying significance at *P ≤ 0.05, **P ≤ 0.01, ***P ≤ 0.001, ****P ≤ 0.0001, and “ns” = P >0.05 (b) Representative phase-contrast micrographs of ∆*lacZYA* cells expressing QueE at the indicated inducer concentration, scale bar = 5 µm. (c-d) Representative Coomassie gel showing protein levels in total cell lysates at different IPTG concentrations and the corresponding western blot showing His_6_-QueE levels. e) APB gel-northern blot detecting tRNA^Tyr^ in total RNA samples of ∆*queE* (SAM31) cells encoding either WT QueE (pRL03), His_6_-QueE (pSY85) or an empty vector (pEB52). Cells were grown in supplemented MinA minimal medium for 2 hours and induced with 0.5 mM IPTG for 3 hours. G-tRNA^Tyr^ = unmodified tRNA^Tyr^, Q-tRNA^Tyr^ = Q-modified tRNA^Tyr^. Note that this panel is a montage with the data for pHis_6_-QueE acquired on another blot, as indicated with a dotted line. (f) Plot showing the relationship between average cell lengths (from panel a) and relative QueE abundance (from panel d), see methods for details.

**Fig (ii). Alanine scanning mutagenesis of amino acid residues in *Ec*QueE important for Q biosynthesis.** (a) APB northern blot detecting tRNA^Tyr^ in total RNA samples of ∆*queE* (SAM31) cells encoding either WT QueE (pRL03), QueE variants, QueE-R27A (pSY98), QueE-C31A (pSA1), QueE-C35A (pSA2.6), QueE-K66A (pSA10), QueE-Q13A (pSY97), QueE-T40A (pSY99), QueE-K60A (pSA9), QueE-S136A (pSA8), QueE-Q189A (pSY100), QueE-C38A (pSA3) or an empty vector (pEB52). Cells were grown in supplemented MinA minimal medium for 2 hours and induced with 0.5 mM IPTG for 3 hours. G-tRNA^Tyr^ = unmodified tRNA^Tyr^, Q-tRNA^Tyr^ = Q-modified tRNA^Tyr^. (b) Representative phase-contrast micrographs of ∆*queE* (SAM31) cells expressing WT QueE and single alanine mutants as indicated above in (a), scale bar = 5 µm. Cells were grown in supplemented MinA minimal medium for 2 hours and induced with 0.5 mM IPTG for 3 hours. (c) Quantification of levels of YFP-tagged QueE and its variants in ∆*queE* (SAM96) cells transformed with plasmids encoding WT YFP-QueE (pSY76), YFP-QueE-E15A (pSA73), YFP-QueE-C31A (pSA75), YFP-QueE-C35A (pSA119), or YFP-QueE-K66A (pSA57). Cells were grown in supplemented MinA minimal medium for 5 hours. Data represent the mean and range from two independent experiments, and fluorescence was quantified from the number of cells (n) per sample as indicated. The red dotted line represents the expression level for WT YFP-QueE, Statistical analysis was done using t-test displaying significance at *P ≤ 0.05, **P ≤ 0.01, ***P ≤ 0.001, ****P ≤ 0.0001, and “ns” = P >0.05. (d) APB gel- northern blot detecting tRNA^Tyr^ in total RNA samples of ∆*queE* (SAM31) cells encoding either WT QueE (pRL03), YFP-QueE (pSY76) or an empty vector (pEB52). Cells were grown in supplemented MinA minimal medium for 2 hours and induced with 0.5 mM IPTG for 3 hours. Cells expressing YFP-QueE were grown in supplemented MinA minimal medium for 5 hours (please see Methods for details on pSY76). G-tRNA^Tyr^ = unmodified tRNA^Tyr^, Q-tRNA^Tyr^ = Q-modified tRNA^Tyr^. Note that this panel is a montage with the data for pYFP-QueE acquired on another blot, as indicated with a dotted line.

**Fig (iii). Analysis of YFP-tagged variants of *E. coli* QueE and orthologs.** (a) Coomassie stain and (b) western blots of cell lysates of *E. coli* strains encoding YFP-tagged *Ec*QueE (pSY76), its variants C31A (pSA75), C35A (pSA119), ∆E45-W67 (pSA76), and QueE orthologs *Bs*QueE (pSA121) and *Pa*QueE (pSA126). Lysates were prepared from cells grown in supplemented MinA minimal medium for 5 hours (please see Methods for details on pSY76 and derivatives). Cells from a strain carrying a genomic *yfp* reporter (SAM54) are indicated as YFP-only control.

**Fig (iv). Q-tRNA formation in cells expressing QueE variants (QueE-C31A and -C35A).** APB gel-northern blot detecting tRNA^Tyr^ in total RNA samples of ∆*queE* (SAM31) cells carrying either an empty vector (pEB52), WT QueE (pRL03), QueE-C31A (pSA1) or QueE-C35A (pSA2). G-tRNA^Tyr^ = unmodified tRNA^Tyr^, Q-tRNA^Tyr^ = Q-modified tRNA^Tyr^. Cells were grown in supplemented MinA minimal medium for 2 hours and induced with 0.5 mM IPTG as indicated (+).

**Fig (v). Sequence analysis and phylogeny of selected QueE orthologs.** (a) Multiple sequence alignment of QueE orthologs from *Bacillus subtilis* str 168, *Pseudomonas aeruginosa* PA01, *Klebsiella pneumoniae*, *Escherichia coli K-12 MG1655*, and *Salmonella Typhimurium* St1408. The cluster binding motif residues are boxed in black, and residues involved in binding S-adenosyl methionine are highlighted using red circles (single residues) and red boxes (sequential residues). Residues involved in substrate binding (CPH_4_/6-CP ligation) are denoted by green rectangles (single residues) and green boxes (sequential residues). A single amino acid residue involved in binding the catalytic magnesium ion is indicated by a purple circle. (b) Phylogenetic tree of the QueE orthologs estimated by the neighbor-joining method (BLOSUM62) using Jalview[5].

**Fig (vi). Analysis of dual functions of *Ec*QueE orthologs in their hosts.** (a) Representative phase-contrast micrographs of *E. coli, Salmonella Typhimurium*, *Klebsiella pneumoniae*, *Pseudomonas aeruginosa*, and *Bacillus subtilis* expressing their native QueE ortholog. The corresponding plasmid constructs are indicated, p*Ec*QueE (pRL03), p*St*QueE (pSA21), p*Kp*QueE (pSA59), p*Pa*QueE (pSA156), pYFP-*Pa*QueE (pSA154). Empty vectors pEB52 for *E. coli*, *S. Typhimurium*, pBAD33 for *K. pnueumoniae*, pPSV38 for *P. aeruginosa*) were included as controls. *B. subtilis* strains encoding *Bs*QueE (*Bs amyE::Bs*QueE, SAA50) and YFP-*Bs*QueE (*Bs amyE::yfp-Bs*QueE, SAA52) at the genomic *amyE* locus are indicated and *B. subtilis* strain carrying empty vector pDR111 sequence at *amyE* locus (*Bs amyE::Empty*, SAA53) was included as a control. All cultures were grown in supplemented MinA minimal medium and QueE expression was induced using 0.5 mM IPTG in all cases, except for *P. aeruginosa* and *B. subtilis* where IPTG was used at 2 mM and 1 mM, respectively. Scale bar = 5µm. (b) Measurement of cell lengths of *B. subtilis* expressing *Bs*QueE (*Bs amyE::Bs*QueE, SAA50), control strain (*Bs amyE::Empty*, SAA53), *P. aeruginosa* cells expressing *Pa*QueE (pSA156), or an empty vector (pPSV38). QueE expression was induced using IPTG at 2 mM and 0.5 mM for *P. aeruginosa* and *B. subtilis*, respectively. Gray circles represent individual cells, mean cell length values are indicated in red, and the horizontal gray bars represent the median. Data are obtained from two independent experiments using (n) number of cells per sample as indicated for each strain, Statistical analysis was done using t-test displaying significance at *P ≤ 0.05, **P ≤ 0.01, ***P ≤ 0.001, ****P ≤ 0.0001, and “ns” = P >0.05. (c) FM 4-64 staining of *B. subtilis* cells expressing either *Bs*QueE (*Bs amyE::Bs*QueE, SAA50) or control strain (*Bs amyE::Empty*, SAA53), QueE expression was induced using 0.5 mM IPTG. Scale bar = 5µm. (d) Quantification of levels of YFP-tagged *Pa*QueE (pSA154) expressed in *P. aeruginosa* with or without induction with 2 mM IPTG, normalized to the background fluorescence in cells carrying an empty vector (pPSV38). Data are obtained from two independent experiments using (n) number of cells per sample as indicated. The corresponding Coomassie-stained gel and western blots show *Pa*QueE protein expression in total cell lysates. (e) Quantification of levels of YFP-tagged *Bs*QueE expressed in *B. subtilis* (Bs *amyE::yfp-Bs*QueE, SAA52) with or without induction with 0.5 mM IPTG, normalized to the background fluorescence in control (*Bs amyE::Empty*, SAA53). Data are obtained from two independent experiments using (n) number of cells per sample as indicated. The corresponding Coomassie-stained gel and western blots show *Bs*QueE protein expression in total cell lysates.

**Fig (vii). Q-tRNA formation in cells expressing QueE deletion mutants (QueE-ΔE45-E51, -ΔV52-W67, and -ΔE45-W67).** APB gel-northern blot detecting tRNA^Tyr^ in total RNA samples of ∆*queE* (SAM31) cells carrying either an empty vector (pEB52), WT QueE (pRL03), QueE- ΔE45-E51 (pSA60), QueE-ΔV52-W67 (pSA61), or QueE-ΔE45-W67 (pSA62). G-tRNA^Tyr^ = unmodified tRNA^Tyr^, Q-tRNA^Tyr^ = Q-modified tRNA^Tyr^. Cells were grown in supplemented MinA minimal medium for 2 hours and induced with 0.5 mM IPTG as indicated (+) for 3 hours.

**Fig (viii). Sequence analysis and phylogeny of selected QueE orthologs from gammaproteobacteria** (a) Multiple sequence alignment of 18 QueE orthologs from representative enterobacteria generated using ClustalW. The E45-W67 region is highlighted in red. (b). Phylogenetic tree of the selected QueE sequences estimated by the neighbor-joining method (BLOSUM62) using Jalview[5].

**Fig (ix). Expression of *Ec*QueE in *P. aeruginosa*.** Representative phase-contrast micrographs of *E. coli* and *P. aeruginosa* cells carrying either p*Ec*QueE (pSA157) or pEmpty (pPSV38 shuttle vector) in the presence or absence of IPTG. IPTG levels of 0.5 mM and 2 mM were used for *E. coli* and *P. aeruginosa*, respectively. Scale bar = 5 µm.

**References**

1. Yadavalli SS, Carey JN, Leibman RS, Chen AI, Stern AM, Roggiani M, et al. Antimicrobial peptides trigger a division block in Escherichia coli through stimulation of a signalling system. Nat Commun. 2016;7: 12340.

2. Bullock WO. XL1-blue : a high efficiency plasmid transforming recA Escherichia coli strain with beta-ga-lactosidase selection. Biotechniques. 1987;5: 376.

3. Galli E, Gerdes K. Spatial resolution of two bacterial cell division proteins: ZapA recruits ZapB to the inner face of the Z-ring. Mol Microbiol. 2010;76: 1514–1526.

4. Amann E, Ochs B, Abel KJ. Tightly regulated tac promoter vectors useful for the expression of unfused and fused proteins in Escherichia coli. Gene. 1988;69: 301–315.

5. Waterhouse AM, Procter JB, Martin D, Clamp M, Barton GJ. Jalview Version 2 - A multiple sequence alignment editor and analysis workbench. Bioinformatics. 2009;25: 1189–1191.
