# Supplementary material for: Queuosine biosynthetic enzyme, QueE moonlights as a cell division regulator
Source: PLoS Genet. 2024 May 20;20(5):e1011287. doi: 10.1371/journal.pgen.1011287 (PMC11142719; doi:10.1371/journal.pgen.1011287)
Supplement: Attachment — Submitted filename: Review of QueE.pdf [file pgen.1011287.s002.pdf]

This manuscript is a follow-up to PMID 27471053. In the prior manuscript it was shown that conditions that induced the PhoPQ system in *E. coli* (sub MIC cationic antimicrobial peptide or low Mg2+ growth in the absence of *mgrB*) results in cell filamentation. This filamentation is dependent on QueE, an enzyme in queosine biosynthetic pathway shown to colocalize with FtsZ and inhibits cell division. In the present manuscript, the authors use a misexpression strategy to genetically separate the enzymatic activity of QueE from the effects of QueE on cell division. They identify a variant of QueE that has functional enzymatic activity and is unable to inhibit cell division and reciprocally, variants of QueE that maintain enzymatic activity in vivo that are unable to inhibit cell division. The also show that the targeting of QueE to FtsZ upon upregulation (when *phoQP* is induced) is generalizable to multiple enterics, but not to more distantly-related organisms like *Bacillus subtilis*. The results represent another example of moonlighting, in which a protein with a characterized function (in this case, modification of GUN tRNAs) possesses at least one other function (in this case, inhibition of cell division). One thing missing from the manuscript that would add value is some indication as to the relationship (including none) of connecting the tRNA modification/1-C pool/etc to cell division. In addition QueE cysteine-to-alanine substitutions which would presumably have reduced ability to form iron-sulfur clusters appear to be fully functional by the assay utilized in the manuscript, yet there is no mention of this fact. The data suggests the iron-sulfur cluster serves a role that is tertiary to both queosine biosynthesis and cell division modulation (at least under the conditions tested); this is actually very interesting and could be more rigorously investigated (in the future) by generating double mutants and/or assessing if the variants actually coordinate the iron-sulfur cluster. Feedback is indicated below.

Feedback

General (optional, but please consider): better if figure legends are not conclusion to be drawn and instead describe assay/etc – lessens chance data/results are conflated with conclusion and leaves responsibility with reader to decide if conclusion in text is supported by data presented.

Fig 1a - The figure would benefit from some tweaking to make clearer. Suggest remove far left QueE cartoon in 1a. Top would be: -PhoQP stress, bottom would be +PhoQP stress (not just any stress induces QueE as is implied in current figure).

Fig 1b – Top: Assays to monitor dual activities of QueE (functions is too strong imo b/c as far as I know, there is no known deterimental phenotype associated with the inability of QueE to filament cells?)

Fig. 1c – one is left wondering what the amount of Q modification is for WT under the growth condition. It does not change any conclusions regarding the complementation but leaves open the question of whether overexpression of the enzyme is necessary for the complementation to work. After going back through several references, I could not identify the copy number of the plasmid being used to let alone get a sense of the amount of expression relative to native using this plasmid/inducible promoter system. It

would be helpful if some sense could be conveyed to the reader as to whether it is w/in 10X or more like 100X, 1000X etc? If it is not known or info cannot be readily provided, this should be acknowledged somewhere as a caveat to interpretation. This is germane to interpreting the LOF variants as increased expression could compensate for reduced functionality not be evident in assay.

Ln 155 – unclear where "exponential" is inferred. The exponential relationship in Fig 2a between IPTG and cell length is not evident. Moreover, there is no direct measurement of induction here (protein expression); in Fig S1d, looks linear-ish up to 250 uM and then plateaus despite increase in cell length seen in Fig 2a quantitation. Needs reworded for accuracy.

Fig S1e – the his-tagged version seems to be cropped from another blot and then added on, but this manipulation is not clearly indicated. There should be some sort of line (dotted etc) showing this is a crop/montage and a statement in the legend indicating the same. Unless equal load on the same blot, direct comparison is inappropriate, so the reader needs to be flagged to not directly compare. The conclusion (has activity) seems fine, but representation of data to reader needs to be more clear.

Ln 176 – are these residue shown to coordinate SAM in the *E. coli* structure or predicted/inferred to do so? Scanning structure paper, it seems SAM is not bound (?), so the inference is from other structures. Confirm what is stated is accurate representation.

Ln 178 – is there any significance to the acetylation sites in the literature? If so, state as it is unclear why they are being investigated.

Fig 2a, S1a etc – what are the boxes in the dot plots? Why are very large shown as actual dots but the rest are not? What are the sample sizes? >50 can be 51 or 10,000, so not very informative when considering reported statistics. When not just state n?

Fig 3b – stylistic, but please consider to help general reader: F/NF and Q/NQ require decoding. What not put column headings as filamentation and Queosine modification then use + or - ? then can class by +/+ -/+ +/- and -/- instead of just amino acid order.

Fig 3d – suggest put controls (2) on top, then mutants on bottom, R27A under empty vector control. Would be easier to compare.

Fig S4 – Indicate what media are these grown in (still MinA?)

Fig 6b – missed queE italic
Ln 343 – has a moonlighting function in modulating FtsZ activity (because may have others funcions that are moonlighting but independent cell division).

Line 440 – missed removing edit

Ln 447 – what is the sequence of the oligos? Did not see in supplementary, but if there, please reference. If not, please include.

Ln 474 – clustal omega citation?

As an aside, it is interesting that like QueE, many of the proteins that modulate FtsZ activity have RE residues in region implicated in FtsZ regulation - see ZipA, ZapD, FtsA, MinCD, SlmA, RefZ. Makes one wonder if EcQueE expressed in Bacillus or Pseudomonas would filament.

I do not make recommendations to journals as to suitability for publication and think journals and/or journal editors should decide suitability for their journal. I select accept with modification by default as there is generally no option to abstain. I sign my reviews to make myself accountable and understand this may become public record.
-Jen Herman
