# Supplementary material for: Queuosine biosynthetic enzyme, QueE moonlights as a cell division regulator
Source: PLoS Genet. 2024 May 20;20(5):e1011287. doi: 10.1371/journal.pgen.1011287 (PMC11142719; doi:10.1371/journal.pgen.1011287)
Supplement: Attachment — Submitted filename: Response to reviewers.pdf [file pgen.1011287.s003.pdf]

We are grateful to all three reviewers for their valuable feedback and suggestions. Our responses to their comments are listed below.

Reviewers' comments:

Reviewer #1:

This manuscript is a follow-up to PMID 27471053. In the prior manuscript it was shown that conditions that induced the PhoPQ system in E. coli (sub MIC cationic antimicrobial peptide or low Mg2+ growth in the absence of mgrB) results in cell filamentation. This filamentation is dependent on QueE, an enzyme in queosine biosynthetic pathway shown to colocalize with FtsZ and inhibits cell division. In the present manuscript, the authors use a misexpression strategy to genetically separate the enzymatic activity of QueE from the effects of QueE on cell division. They identify a variant of QueE that has functional enzymatic activity and is unable to inhibit cell division and reciprocally, variants of QueE that maintain enzymatic activity in vivo that are unable to inhibit cell division. The also show that the targeting of QueE to FtsZ upon upregulation (when phoQP is induced) is generalizable to multiple enterics, but not to more distantly-related organisms like Bacillus subtilis. The results represent another example of moonlighting, in which a protein with a characterized function (in this case, modification of GUN tRNAs) possesses at least one other function (in this case, inhibition of cell division). One thing missing from the manuscript that would add value is some indication as to the relationship (including none) of connecting the tRNA modification/1-C pool/etc to cell division. In addition QueE cysteine-to-alanine substitutions which would presumably have reduced ability to form iron-sulfur clusters appear to be fully functional by the assay utilized in the manuscript, yet there is no mention of this fact. The data suggests the iron-sulfur cluster serves a role that is tertiary to both queosine biosynthesis and cell division modulation (at least under the conditions tested); this is actually very interesting and could be more rigorously investigated (in the future) by generating double mutants and/or assessing if the variants actually coordinate the iron-sulfur cluster. Feedback is indicated below.

Feedback
General (optional, but please consider): better if figure legends are not conclusion to be drawn and instead describe assay/etc – lessens chance data/results are conflated with conclusion and leaves responsibility with reader to decide if conclusion in text is supported by data presented.
We thank Dr. Herman for this general comment. It was initially a stylistic choice made by the authors to narrate the paper to include conclusions as the figure legends. Based on the reviewer's feedback, we have made changes to all of the main figure legends such that they do not describe the conclusion.

Fig 1a - The figure would benefit from some tweaking to make clearer. Suggest remove far left QueE cartoon in 1a. Top would be: -PhoQP stress, bottom would be +PhoQP stress (not just any stress induces QueE as is implied in current figure).
We have made changes to Fig 1a and its legend to clarify that QueE plays a role under PhoQP activating stress conditions.

Fig 1b – Top: Assays to monitor dual activities of QueE (functions is too strong imo b/c

as far as I know, there is no known deterimental phenotype associated with the inability of QueE to filament cells?)

We agree that a phenotype has not yet been identified for cells unable to filament. However, there are several proteins whose function is believed to be known or well-characterized, but upon deletion/ mutation do not display a detrimental phenotype. So here we would like to clarify that we are referring only to a protein's secondary or alternate function. As per the reviewer's suggestion, we have replaced the word "functions" with "activities" in Fig 1b and its legend.

Fig. 1c – one is left wondering what the amount of Q modification is for WT under the growth condition. It does not change any conclusions regarding the complementation but leaves open the question of whether overexpression of the enzyme is necessary for the complementation to work. After going back through several references, I could not identify the copy number of the plasmid being used to let alone get a sense of the amount of expression relative to native using this plasmid/inducible promoter system. It would be helpful if some sense could be conveyed to the reader as to whether it is w/in 10X or more like 100X, 1000X etc? If it is not known or info cannot be readily provided, this should be acknowledged somewhere as a caveat to interpretation. This is germane to interpreting the LOF variants as increased expression could compensate for reduced functionality not be evident in assay.

We thank Dr. Herman for pointing out the confusing aspects of the plasmid expression system. Firstly, all the plasmids used to test QueE expression in *E. coli* are derivatives of pTrc99a (originally described in Amann E et al., 1988). pTrc plasmids are considered medium copy with a copy number of ~30 per cell and they carry *lacI*^{q}, which represses the lac promoter reducing leaky expression. We have added this information about the plasmids to the Methods section. Secondly, we would like to address the key question raised here, whether overexpression of the enzyme is necessary for the complementation of Q-biosynthesis. In this revision, we have added data to Figure 1c showing that overexpression of QueE is not necessary to complement Q-biosynthetic activity. We have rewritten the first section of the results to describe this result. In addition, as the reviewer mentioned, it is possible that the loss of function variants may be catalytically less active than wild-type QueE so we performed APB-northern blots for cells expressing the variants with or without induction. We have added these data (please see Figures S4, and S7 in the revised manuscript) and updated the Results section accordingly.

Ln 155 – unclear where "exponential" is inferred. The exponential relationship in Fig 2a between IPTG and cell length is not evident. Moreover, there is no direct measurement of induction here (protein expression); in Fig S1d, looks linear-ish up to 250 uM and then plateaus despite increase in cell length seen in Fig 2a quantitation. Needs reworded for accuracy.

We are sorry for this confusion, and we have replaced "exponential" with "linear".

Fig S1e – the his-tagged version seems to be cropped from another blot and then added on, but this manipulation is not clearly indicated. There should be some sort of line (dotted etc) showing this is a crop/montage and a statement in the legend indicating the same. Unless equal load on the same blot, direct comparison is inappropriate, so

the reader needs to be flagged to not directly compare. The conclusion (has activity) seems fine, but representation of data to reader needs to be more clear.
We thank the reviewer for catching this oversight. Yes, the data for His-tagged version was cropped to avoid confusion to the reader, as it was part of a much bigger gel with a group of unrelated samples. To clarify this, we have added a dotted line separating this sample and explained it in the legend, and as the reviewer rightly noted, this does not affect the conclusion that this construct has Q biosynthesis activity.

Ln 176 – are these residue shown to coordinate SAM in the E. coli structure or predicted/inferred to do so? Scanning structure paper, it seems SAM is not bound (?), so the inference is from other structures. Confirm what is stated is accurate representation.
Yes, this is correct. These residues are highly conserved and are shown to bind either SAM or a SAM-derived adduct in other QueE orthologs whose structures have been resolved. Accordingly, we have rephrased this sentence to say that these residues are predicted to bind SAM.

Ln 178 – is there any significance to the acetylation sites in the literature? If so, state as it is unclear why they are being investigated.
Based on the literature, lysine acetylation allows for modulation of protein activity (VanDrisse, Chelsey M., and Jorge C. Escalante-Semerena. 2019. "Protein Acetylation in Bacteria." ***Annual Review of Microbiology*** **73 (September): 111–32). We have rephrased the sentence to clarify why the lysines are included in the analysis.**

Fig 2a, S1a etc – what are the boxes in the dot plots? Why are very large shown as actual dots but the rest are not? What are the sample sizes? >50 can be 51 or 10,000, so not very informative when considering reported statistics. When not just state n?
The box represents the middle 50% of scores for the group. The range of scores from lower to upper quartile is referred to as the inter-quartile range. The middle 50% of scores fall within the inter-quartile range. Seventy-five percent of the scores fall below the upper quartile. The dots represent values outside the range, closer to the minimum and maximum value of the data set. To simplify these figures showing cell lengths, we have now replaced the box plots with dot plots, showing all data points as circles (please see Figures 2, 4, S1, and S6). We have also indicated the sample sizes (the exact number of cells analyzed) in all plots showing data for cell lengths or fluorescence quantification (please see Figures 2, 4, 5, 6, S1, S2, and S6).

Fig 3b – stylistic, but please consider to help general reader: F/NF and Q/NQ require decoding. What not put column headings as filamentation and Queosine modification then use + or - ? then can class by +/+ -/+ +/- and -/- instead of just amino acid order.
We thank Dr. Herman for her suggestions to make this table easy to read. As suggested, we replaced "cell morphology" with "filamentation" and used "+" and "-" to indicate the effects on filamentation and Q biosynthesis. We tried to classify the information by +/+ -/+

+/- and -/-, however, after trying a few different ways, we left the amino acids in order for simplicity.

Fig 3d – suggest put controls (2) on top, then mutants on bottom, R27A under empty vector control. Would be easier to compare.
We have rearranged the images for easier comparison of mutants with the controls.

Fig S4 – Indicate what media are these grown in (still MinA?)
Yes, the cells were grown in supplemented MinA minimal medium. We have updated the figure legend with the media and growth conditions used (please see Figure S6 in the revised submission).

Fig 6b – missed queE italic
Thanks for catching the error, we have fixed it.

Ln 343 – has a moonlighting function in modulating FtsZ activity (because may have others funcions that are moonlighting but independent cell division).
We have added a phrase to this sentence to say "…moonlighting function in modulating cell division". Based on previous work, it appears that FtsZ ring formation is not affected in filaments, and it is unclear if FtsZ activity is altered. Our ongoing research is focused on understanding at which stage QueE disrupts division and which protein(s) it interacts with.

Line 440 – missed removing edit
Thanks, we have fixed the error.

Ln 447 – what is the sequence of the oligos? Did not see in supplementary, but if there, please reference. If not, please include.
The oligonucleotide sequence is mentioned in Table C in supporting information (Oligo SA1). As suggested, we have added a reference, so it is easy to locate.

Ln 474 – clustal omega citation?
Thanks, we added the missing citation.

As an aside, it is interesting that like QueE, many of the proteins that modulate FtsZ activity have RE residues in region implicated in FtsZ regulation - see ZipA, ZapD, FtsA, MinCD, SlmA, RefZ. Makes one wonder if EcQueE expressed in Bacillus or Pseudomonas would filament.
We thank Dr. Herman for bringing up this interesting point. While we do not yet fully understand if/how QueE modulates FtsZ activity (or another protein in the cell division pathway), it is interesting to see if *Ec*QueE expressed in Bacillus or Pseudomonas would

cause filamentation. To test this, we expressed *Ec*QueE on a shuttle vector in both *E. coli* and *P. aeruginosa*. Interestingly, we do not see any filamentation in *P. aeruginosa* but our control *E. coli* cells display filaments. This result indicates that in addition to the sequence and structural differences between the two QueE orthologs, there are likely additional factors and variations in cell division machinery in Pseudomonas leading to this outcome. We have added this data to the supporting information (please see Figure S9 in the revised manuscript) and added the Result to the Discussion section.

I do not make recommendations to journals as to suitability for publication and think journals and/or journal editors should decide suitability for their journal. I select accept with modification by default as there is generally no option to abstain. I sign my reviews to make myself accountable and understand this may become public record.
-Jen Herman

<u>Reviewer #2:</u>

This paper from Adeleye and Yadavalli investigates the cell division inhibition activity of Escherichia coli QueE, an enzyme involved in Queuosine (Q) synthesis for tRNA modification. The authors set out to determine whether the roles of QueE in division inhibition and Q synthesis are separable and whether this activity is conserved. Site-directed mutagenesis was performed to generate variants of QueE with amino acid substitutions in the active site region and other areas of the protein, including a region implicated in cell division by bioinformatic analysis. Plasmids expressing the altered proteins were then tested for their ability to promote Q biosynthesis and division inhibition using blotting and microscopy, respectively. The authors also overexpressed QueE orthologs from other bacteria in E. coli and in their native hosts to test for conservation of the division inhibition activity. Close relatives of E. coli produce QueE proteins that block division, whereas more distant relatives (P. aeruginosa and B. subtilis) do not. Overall, the results support a moonlighting function in division inhibition for QueE that is independent of catalytic activity needed for Q synthesis.

The paper is very straightforward, and results clearly presented and described. In general, I agree with the conclusions and that they are supported by the data. However, there are some areas of the paper that would benefit from revision.

Major points:

1) The QueE variants that do not block cell division were tested for stable accumulation based on the fluorescence of YFP fusions. This assay is not sufficient, as the fusion protein could be cleaved with the YFP portion stably accumulating and the QueE portion being degraded. Immunoblots should be performed to ensure that full-length protein for these variants accumulates to levels similar to that of the WT protein.
We thank the reviewer for this valid concern. Based on their suggestion, we have performed western blots to show that the full-length proteins for the variants accumulate similar to the WT protein (please see Figure S3 in the revised manuscript).

2) I think it would be worth trying AlphaFold to see if it predicts an interaction between Ec-QueE and Ec-FtsZ and whether the residues in this study implicated in division inhibition lie at the

putative interface. If results for this analysis were positive, it would significantly strengthen the paper.

We thank the reviewer for this interesting suggestion. We tried Alphafold-multimer to analyze interactions between *Ec*QueE and *Ec*FtsZ, however, the software did not predict an interaction between the two proteins. This result appears to be consistent with our observation that FtsZ ring formation is not affected in filamenting cells, and it is unclear if FtsZ is a target of QueE at the division site. Our ongoing research is focused on understanding at which stage QueE disrupts division and which protein(s) it interacts with.

Minor points:

3) Given the number of proteins with moonlighting functions that have now been characterized, I do not think it is necessary to make a major point about challenging the one enzyme, one function concept in the Author Summary.

According to the reviewer's suggestion, we have removed the phrase "one enzyme, one function" from the Author summary".

4) Lines 116-122 are redundant with the Introduction section and could be cut.

We thank the reviewer for pointing out this redundancy, these lines have been removed.

5) Line 679: QueE regulates… --> QueE inhibits…

The Figure 2 legend has been reworded based on the reviewer 1's suggestion such that it does not draw a conclusion to the reader.

6) Figure 2b – increase size of labels.

We have rearranged the two panels and increased the font size on the labels in Fig 2b.

7) Figure 3 legend. Amino acid residues are "substituted" not "mutated". Mutation is a term used for genes not proteins.

We have replaced "Mutation" with "Substitution" in the Figure 3 legend.

8) Fig. 3A: Add color to the AAs shown as sticks to make them stand out.

We have color-coded the relevant residues (R27, C31, and C35) to make them stand out.

9) Figure 3B: The first column would be better labeled as "Amino acid substitution", and the entries as "Q13A, E15A, etc….".

We thank the reviewer for this helpful suggestion. Accordingly, we have made the changes to Fig 3b for accuracy.

10) Fig. 4A: The cells are way too small to see the Z-rings and QueE localization. An inset with a zoom is needed.

Thanks to the reviewer for pointing this out. We have revised this figure by enlarging this panel to better visualize the Z-rings.

11) Fig 4B: Is QueE in these constructs produced from a gene under different regulation that the IPTG-induced plasmids used in other panels? If so, this should be explicitly indicated.
The constructs used here were IPTG-induced. Based on the reviewer's suggestion, we have added this information to the figure legend.

<u>Reviewer #3:</u>

This study by Adeleye and Yadavalli illuminates the contribution of the conserved tRNA modification enzyme QueE to division inhibition in E. coli. Induced in response to cationic antimicrobial peptides (CAP) or magnesium starvation via PhoP/Q, accumulation of the queE gene product leads to filamentation presumably through interaction with components of the divison machinery. QueE colocalizes to the future division site with the conserved bacterial division protein FtsZ. Through a combination of biochemistry, genetics, and comparative genomics, they identify a domain of E. coli QueE that is involved in mediating division inhibition independent of QueE's role in tRNA modification. Additionally, they provide data suggesting that while QueE overproduction leads to filamentation in E. coli and closely related organisms, it does not modulate division in other bacteria including Pseudomonas and Bacillus subtilis.

While the physiological relevance of having two, apparently disparate functions encoded by a single protein remains unclear, I found experiments and computational analysis indicating that two, separate domains are involved in QueE mediated tRNA modification and division inhibition very compelling.
At the same time, the work would benefit from additional quantitative data on queE/QueE expression/accumulation in various conditions including antimicrobial challenge and magnesium starvation. Although the authors provide a (single?) quantitative immunoblot indicating His-QueE fusion protein levels at different IPTG concentrations (Figure S1) and relative fluorescence data from YFP-QueE-WT and various mutant constructs (Figures 4 and S2), how QueE concentrations following CAP treatment or magnesium starvation compare to those in strains encoding IPTG inducible queE constructs is unclear.
We agree with the insightful comments made by the reviewer. It would be ideal to quantify the amount of QueE protein in cells grown in different conditions (treatment with antimicrobial peptide, magnesium starvation, other PhoQP-activating conditions as well as plasmid expression). Unfortunately, an antibody against *Ec*QueE is not available and it is quite challenging to estimate absolute concentrations of this protein. Therefore, we have had to rely on epitope-tagged constructs. To best address the reviewer's comment, we spent a substantial amount of time and effort to make genomic constructs carrying an N-terminal epitope-tagged QueE at its native locus, however, our attempts to engineer these strains have failed so far. We tried to get the construct required for recombineering synthesized by a commercial vendor and they failed to generate it after weeks of trials. It appears that these constructs are technically challenging to create, and it is also possible that the locus is less amenable to genetic manipulation.

Despite the above, we have cell length data for cells grown in different conditions that lead to filamentation: 1) wild-type cells treated with antimicrobial peptide, 2) *ΔmgrB* cells grown in magnesium starvation, and 3) plasmid expression. In our experience, average cell lengths

correlate quite well with the level of QueE expression. Data for conditions 1) and 2) were previously published (Yadavalli *et al.,* 2016); under these filamenting conditions, the cell lengths can be as high as 100-200 µm with averages of ~17 µm and 24 µm, respectively. Data for condition 3) is derived from the current work and the average cell length for plasmid-encoded QueE at the highest induction level (0.5 mM IPTG) is ~25 µm. So, the cell lengths for filamenting cells overexpressing QueE on a plasmid are comparable to the measurements obtained under physiological conditions where cells upregulate QueE. We have added text to include this explanation in the Results section to further support our experimental setup and findings.

Moreover, without information on QueE concentration it is impossible to assess whether queE overexpression really has no impact on B. subtilis or P. aeruginosa division, or if levels are too low to impact division in those organisms. The addition of this information would enhance experimental rigor and provide physiological benchmarks with which to assess the results.
The reviewer raises a critical issue of whether the *B. subtilis* or *P. aeruginosa* QueE expression is too low to impact division in these organisms. To address this question, we took the following steps -

- **For expression in *B. subtilis*: Because *B. subtilis* is known to display plasmid instability/ maintenance issues, we decided to generate genomic constructs of *B. subtilis* which carry inducible *Bs*queE. With help and guidance from Dr. David Dubnau and Dr. Jeanette Hahn (Rutgers-Newark), who are experts in *B. subtilis* genetics, we generated *B. subtilis* strains containing IPTG-inducible *Bs queE*, *yfp-Bs queE*, and a control strain carrying empty vector pDR111 sequence at the *amyE* locus (strains SAA50, SAA52, and SAA53 in Table A, SI). These constructs have been designed to ensure optimal expression in *B. subtilis*, including a ribosome binding site chosen from a well-expressed competence protein.**
- **For expression *P. aeruginosa*: To ensure robust expression in *P. aeruginosa*, we reached out to Dr. Simon Dove (Boston Children's Hospital, Harvard Medical School) and obtained an expression vector that has been widely used for efficient protein expression in Pseudomonas. We cloned *Pa*QueE and YFP-*Pa*QueE in this vector (pSA156 and pSA154 in Table B, SI) to evaluate the expression as well as the two QueE activities.**

Using the constructs described above, we 1) performed microscopy to visualize cell morphology and analyze cell lengths, 2) measured the accumulation of YFP-tagged QueE, and 3) performed western blot analysis on cultures uninduced and induced for QueE expression to confirm the full-length product (Please see Figure S6 and the updated Results section in the revision). Overall, we see a strong induction and expression of *Bs*QueE in *B. subtilis* and *Pa*QueE in *P. aeruginosa* with negligible impact on cell division.

Other comments:
1. Line 129: Please clarify whether or not queE is induced in some manner here and other places in the text.
We apologize that we forgot to include the details of induction here. Yes, plasmid-encoded QueE was induced using IPTG here and the details are also included in the methods. In the revised manuscript, we have clarified this part and referenced the Methods section for further details on growth conditions.

2. Line 136. This section is not particularly clear. It is presented as results but is more generally an explanation of the assays. It would be better to fold into the next section which tests the dose-dependency of QueE on division

We thank the reviewer for their input. We have re-written this section in the revision keeping the explanation of assays to a minimum and focusing on the level of QueE required for Q-biosynthesis.

3. Line 207: How high do levels need to be to cause filamentation? (i.e. the meaning of 'high' is unclear—5 times basal levels? 100x basal levels?)

We thank the reviewer for asking to clarify the term 'high' here. Based on our data from Figures 2 and S1, QueE levels need to be about 3x or higher than the basal (uninduced) level of expression. We have now added text to be more precise about what high level refers to.

4. Bacillus subtilis typically grows as a mixture of short and long chains of separated cells. In order to accurately evaluate the impact of BsQueE on division, it is important to use a cell wall stain to resolve septa between individual bacteria.

Yes, we completely agree with the reviewer and there may be partial or complete septa formed between cells within a chain of *B. subtilis* cells. If there are any invaginations of the cell wall and the underlying cytoplasmic membrane, we should be able to visualize them using a stain. We used a membrane stain, FM4-64 to analyze if there were any qualitative differences in septal formation between cells expressing BsQueE and control cells that do not express this protein. Based on our data, there are no significant differences in partial or complete septa formed between the two cell types, indicating that BsQueE may have a negligible to no impact on cell division in *B. subtilis*. We have updated the Results section accordingly and added a corresponding Figure S6c in the revised manuscript.
